# Cognitive behavioral therapy for insomnia in a military traumatic brain injury clinic: a quality improvement project assessing the integration of a smartphone application with behavioral treatment

**DOI:** 10.3389/frsle.2023.1268967

**Published:** 2023-12-19

**Authors:** Justin T. Matsuura, Nicole S. Keller, Michael B. Lustik, Carmen E. Campbell, Chad E. Grills

**Affiliations:** ^1^Department of Rehabilitation Services, Interdisciplinary Pain Management Center, Tripler Army Medical Center, Tripler AMC, HI, United States; ^2^Department of Clinical Investigation, Tripler Army Medical Center, Tripler AMC, HI, United States; ^3^Brain Injury Clinic, Desmond T. Doss Health Clinic, Schofield Barracks, HI, United States

**Keywords:** insomnia, brain injury, US military health system beneficiaries, cognitive behavioral therapy, smartphone app, rehabilitation

## Abstract

**Objectives:**

While the association between insomnia and traumatic brain injury (TBI) is well established, TBI rehabilitation programs that focus on sleep as a primary target are limited. Cognitive behavioral therapy for insomnia (CBTi) is an effective treatment for insomnia, however; its use within TBI clinics is relatively unknown. Therefore, our aim was to evaluate the implementation of CBTi, used in conjunction with a smartphone app for insomnia, within a US military TBI program to improve care within this setting.

**Setting:**

A TBI clinic at a US military installation.

**Methods:**

MHS beneficiaries underwent 6 sessions of CBTi and a 1-month post-treatment follow up session. Data was collected at each treatment session as part of routine clinical care.

**Results:**

A total of 69 US MHS beneficiaries seen at a TBI clinic with a diagnosis of insomnia began CBTi. Attrition rate at the end of the CBTi program and 1-month posttreatment session was 35% and 48%, respectively. Results demonstrated that sleep onset latency (SOL) and wake after sleep onset (WASO) decreased during treatment (*p*'s < 0.001). Further, symptoms reported on the Insomnia Severity Index (ISI) improved during CBTi (*p* < 0.001).

**Conclusion:**

Findings demonstrate how CBTi used in conjunction with a CBTi smartphone application can be used to effectively treat insomnia for MHS beneficiaries seeking care for TBIs. This evaluation provides the basis for further research on how CBTi may improve care within TBI programs.

## 1 Introduction

Traumatic Brain Injuries (TBIs), primarily because of blast exposure, were the “signature wound” associated with Operation Enduring Freedom (OEF) in Afghanistan and Operation Iraqi Freedom (OIF) (Cifu et al., 2010). Since 2000, there have been 468,424 TBIs sustained by service members (SMs), which include significant increases in injuries during OEF and OIF, resulting in a surge in demand for adequate acute and extended follow up medical services (Swanson et al., 2017; Health.mil, 2023). TBIs, particularly mild traumatic brain injuries (mTBIs), resulting from blast related injuries, have been found to be associated with a multitude of medical (e.g., headaches, obstructive sleep apnea) and psychiatric concerns (e.g., PTSD, insomnia) (Cifu et al., 2010; Collen et al., 2012; Mysliwiec et al., 2013).

Insomnia is one of the most frequently co-occurring conditions associated with TBIs, impacting ~30–50% of these patients (Mathias and Alvaro, 2012; Dietch and Furst, 2020). Insomnia is conceptualized as a “dissatisfaction with sleep quantity and quality” during sleep initiation and sleep maintenance (American Psychiatric Association, 2022). The prevalence of insomnia is higher for military populations who have sustained TBIs compared to those who have not (Epstein et al., 2012; Mosti et al., 2019). In addition, insomnia associated with TBIs negatively impacts medical recovery and decreases the likelihood of SMs returning to full duty (Mollayeva et al., 2020). Further, insomnia is associated with poorer recovery from comorbid behavioral health conditions (e.g., PTSD) and impairments in cognitive functioning (e.g., reaction time) (Troxel et al., 2015). Although there is a critical need for effective treatments to improve sleep in TBI clinics, many TBI treatment programs servicing MHS beneficiaries (i.e., SMs, family members, retirees, veterans) do not focus on sleep as a primary treatment target (Gilbert et al., 2015).

Cognitive behavioral therapy for insomnia (CBTi) is recognized as an effective treatment for chronic insomnia (Kuhn et al., 2016). Research indicates that between 70 and 80% of patients with insomnia experience sustained benefits from CBTi and ~50% experience clinical remission ((Dietch and Furst, 2020). Further, CBTi has been found to improve sleep among specific populations including SMs (Taylor et al., 2017; Lee et al., 2021). CBTi is a manualized treatment and typically includes the following: sleep education, sleep hygiene, relaxation training, sleep restriction, stimulus control, and cognitive restructuring (Ouellet and Morin, 2004).

While TBIs may often result in patients experiencing symptoms of insomnia, the effectiveness of CBTi within TBI clinics are relatively unknown as few such studies have been conducted to examine its use with this population (Ludwig et al., 2020). Ludwig et al. (2020) conducted a comprehensive review of studies that evaluated the use of CBTi among individuals with a TBI. Most studies that were included in their evaluation (3 of 4) were case studies or case series that found that CBTi improved reported sleep behaviors or symptoms comorbid with insomnia (Ouellet and Morin, 2004, 2007; Lu et al., 2016). Theadom et al. (2018) conducted a pilot randomized controlled study comparing online programs, specifically CBT skills for sleep to an educational program, in which the CBT program demonstrated greater improvements in self-reported sleep quality compared to the educational program. Therefore, although promising, the implementation of CBTi as part of routine care in TBI programs should be further evaluated to better understand its effectiveness with this population.

Although CBTi has been found to be an effective treatment for sleep problems among military personnel (Taylor et al., 2017; Lee et al., 2021), special considerations may need to be taken into account for patients being treated in TBI clinics (Gallagher et al., 2016). Research suggests sleep difficulties may have greater impact on cognitive functioning among those with TBI (Bloomfield et al., 2010). In addition, cognitive dysfunction such as deficits in working memory, episodic memory, attention, and executive function may present as barriers to effective CBTi implementation with individuals who have sustained TBIs (Dietch and Furst, 2020). For example, CBTi is a treatment that uses skills that involve memory and executive functioning (e.g., remembering to attend appointments, the completion of take-home tasks, and following directions). Currently, it is unclear whether cognitive deficits would adversely impact treatment for patients who sustained TBIs. Adaptations to CBTi delivery for patients being seen in TBI clinics may be needed to optimize treatments (Dietch and Furst, 2020). For example, preliminary evidence suggests smartphone apps may help to improve behavioral treatment adherence and understanding of treatment materials among patients who have sustained moderate-to-severe TBIs (Rabinowitz et al., 2022).

Smartphone apps offer certain advantages to improve treatment adherence and treatment effectiveness by providing patients with a highly convenient way to access treatment materials and complete at-home assignments independently (Cavanagh et al., 2020). Specifically, research has found that smartphone apps have been effective in helping patients make positive behavioral changes such as adhering to specific medical treatment regimens, making healthy lifestyle choices, and increasing self-efficacy (Lunde et al., 2018; Kazemi et al., 2022). To address sleep difficulties among MHS beneficiaries, the Veteran's Affairs (VA), Stanford University, and Department of Defense (DOD) developed CBTi Coach to support patient adherence to clinical treatment by utilizing features such as a sleep diary tracker, reminders, and checklists (Koffel et al., 2018). CBTi Coach promotes daily interactions and sleep diary completion through key features such as checklists and reminders (Kuhn et al., 2016). Despite having several promising features to improve treatment adherence and sleep in general, little is known as to how effective a CBTi program, which utilizes CBTi Coach, would be for MHS beneficiaries seeking care within TBI clinics.

Research on the effectiveness of CBTi Coach has been promising, albeit somewhat limited. Currently, only one pilot study has evaluated the effectiveness of CBTi Coach which found the app to be effective in decreasing reported symptoms of insomnia among veterans as a standalone treatment (Reilly et al., 2021). Further, Insomnia Coach, an app that functions as a fully automated and self-directed clinical program to address symptoms of insomnia was recently developed by the VA (Kuhn et al., 2019). Although interventions may be more accessible and convenient when using Insomnia Coach, treatment efficacy on the app is currently limited and face-to-face empirically based behavioral treatments remain as standard of care practices within the DOD (U.S. Department of Veterans Affairs and Department of Defense., 2019; Kuhn et al., 2022).

Therefore, our aim is to describe how an in-person CBTi protocol was implemented in the context of a multidisciplinary clinic for TBIs. Importantly, we will also describe how CBTi Coach can be utilized as a treatment aid to improve patient care. Further, we will provide initial pilot data on the utility of CBTi used in conjunction with CBTi Coach for MHS beneficiaries being seen in a TBI clinic. Observations on adherence to the CBTi treatment program are also discussed and recommendations for future research on CBTi for those who sustained TBIs are described.

## 2 Method

This evaluation was reviewed and approved as a quality assurance/quality improvement project by the Tripler Army Medical Center quality assurance/quality improvement review board and the Deputy Commander for Quality and Safety at Tripler Army Medical Center. Data was collected during patients' treatment sessions and documented in their medical record as part of routine clinical care.

### 2.1 Patients

Patients were US MHS beneficiaries (i.e., SMs, family members, retirees) aged 18 or older diagnosed with chronic insomnia disorder or unspecified insomnia disorder per the Diagnostic and Statistical Manual of Mental Disorders – Fifth Edition (DSM-V) criteria through a semi-structured interview (American Psychiatric Association, 2013). MHS beneficiaries were evaluated and treated at a TBI clinic at a US military installation. Patients were initially seen by the TBI primary care manager, who subsequently referred each patient to medical subspecialties (e.g., clinical psychology) based on their initial assessment. Patients with medical or psychological comorbidities, as well as those who were using psychotropic medications for sleep were included in the analyses. Patients who were included in this study were seen in the clinic as part of their routine clinical care.

### 2.2 Cognitive behavioral therapy for insomnia

Patients completed a six-week, in-person, CBTi treatment program (weekly individual sessions) and attended a 1 month post-treatment follow up session to reinforce learned skills (week 10). The CBTi program was part of clinical psychology services offered within the TBI clinic. The program was informed by existing treatment manuals (Teasdale et al., 2013; Edinger and Carney, 2014; Manber et al., 2014)^1^. The CBTi program was implemented as follows: Week 1 (sleep education), week 2 (relaxation training), week 3 (sleep restriction/stimulus control), week 4 (cognitive therapy), week 5 (mindfulness), week 6 (skills review and relapse prevention).

### 2.3 CBTi coach

The CBTi Coach app for iOS or Android platforms was used by patients depending on what mobile service provider they were associated with Hoffman et al. (2013a,b). Both versions of the app are licensed software owned by the VA. Features on the app are used to increase patient engagement in CBTi and improve adherence to treatment (Kuhn et al., 2016). Patients were instructed to use features on the app to complete sleep diaries and practice relaxation exercises. The sleep diary feature included questions to obtain self-reported sleep behavior data such as total sleep time, sleep onset latency, wake after sleep onset.

### 2.4 Measures

*Behavioral sleep measures*. All patients were asked to complete a sleep diary throughout treatment. Sleep behavior data collected at each session included: total sleep time (TST), sleep onset latency (SOL), and wake after sleep onset (WASO). Patients input sleep diary data either using the CBTi Coach app or using a pencil-and-paper sleep diary. Sleep dairy data from each night was obtained by a CBTi clinician and mean scores were calculated for TST, SOL, and WASO and recorded as weekly averages for this project.

*Insomnia severity index (ISI)* is a 7-item self-report questionnaire used to assess the severity of sleep related difficulties (Morin, 1993). Items on the ISI assess the following: Severity of sleep onset and maintenance, satisfaction/dissatisfaction with current sleep pattern, interference with daily functioning due to sleep problems, how noticeable sleep problems are to others, and sleep related distress. Responses are evaluated using a 4-point Likert scale (response descriptions vary per item). Items are summed for a total score (total score range from 0 to 28). The ISI was administered at each session as part of routine clinical care.

*Epworth sleepiness scale* (ESS) is an 8-item questionnaire that is used to evaluate daytime sleepiness in adults (Johns, 1991). Items are presented as eight ordinary life situations (e.g., watching TV) and users rate their likelihood of sleeping in these situations on a 4-point Likert scale (0 = No chance of dozing – 3 = high chance of dozing). Items are summed for a total score (range from 0 to 24). The ESS was administered at each session as part of routine clinical care.

### 2.5 Statistical analyses

Daily sleep diary data was computed into mean scores for each week. Total scores for ISI and ESS were computed each week. Repeated measures mixed effects analyses were used to examine changes in mean levels of treatment outcome data over time, followed by pairwise comparisons among time points if the overall effect of time was significant. Table 1 presents summary statistics based on raw data for each variable. Because data for SOL and WASO were highly skewed, for analysis these two variables were transformed to the log scale to enhance normality and transformed back to the original scale and presented as geometric means in the text. A significance level of 0.05 was used for all analyses, with a Tukey Kramer adjustment to account for multiple comparisons among time points. Error bars in charts represent 95% confidence intervals on the means.

**Table 1:** Summary statistics based on raw data for each variable.

	**Week 1**			**Week 6**			**Week 10**		
**Variable**	**n**	**Mean**	**SD**	**n**	**Mean**	**SD**	**n**	**Mean**	**SD**
**Sleep Diary Variables**									
SOL	54	50.2	40.7	42	29.0^*^	24.9	24	27.1^*^	19.7
WASO	55	41.8	35.2	42	29.0^*^	30.6	24	30.8	34.6
TST	55	339.2	67.8	42	329.7	80.2	24	339.3	79.1
**Questionnaire Data**									
ISI	69	16.7	4.5	45	12.5^*^	6.3	36	12.1^*^	7.0
ESS	69	10.5	5.3	45	9.3	5.8	36	9.0	6.1

^*^P-value < 0.05 for pairwise comparison with week 1.

## 3 Pilot intervention findings

### 3.1 Patient characteristics

A total of 69 US MHS beneficiaries, seen at a TBI clinic with a diagnosis of insomnia, were included in the evaluation. At the time of data analysis 45 patients (65% of total patients) had completed the full 6 six-week session CBTi protocol and 36 patients (52% of total patients) completed the 1-month post-treatment follow up session. All 36 who came to the week-10 follow-up provided data for ISI and ESS, but only 24 provided data on SOL, WASO, and TST.

Of the 69 patients, 50 (72%) had a history of TBI, including mTBI (33 with 1 TBI, 11 with 2 TBIs, 5 with > 2 TBIs, and 1 unknown number of TBIs). Use of sleep medications was recorded during treatment. At initial evaluation, 41 patients were taking sleep medications (59%), compared to 16 of 42 patients at session 6 (38%) and 16 of 36 patients at 1-month post-treatment follow up (44%).

In terms of sleep diary completion, 55 out of 68 (81%) patients had completed their sleep diary at session 1/week 1 (54 used CBTi Coach, 1 used pencil and paper). At the 1 month follow up session, 24 out of 36 (67%) patients continued to complete their sleep diary (23 used CBTi Coach, 1 pencil and paper).

## 4 Results

### 4.1 Sleep diary variables: TST, SOL, WASO

For sleep diary variables, repeated measures analyses found that mean levels of SOL and WASO differed significantly over time (*p* < 0.001 for both). Pairwise comparisons showed lower mean levels at weeks 6 (treatment end) and 10 (1 month follow-up) compared to the initial treatment session (geometric mean SOL = 39 min, 21 min, and 17 min at weeks 1, 6, and 10, respectively, *p* = 0.001 for week 1 vs. week 6 comparison, and *p* < 0.001 for week 1 vs. week 10; geometric mean WASO = 31 min, 18 min, and 21 min at weeks 1, 6, and 10, respectively, *p* = 0.016 for week 1 vs. week 6, and *p* = 0.322 for week 1 vs. week 10).

There was no significant difference in mean levels for TST over time.

### 4.2 Questionnaire data: ISI, ESS

**Figure 1:**
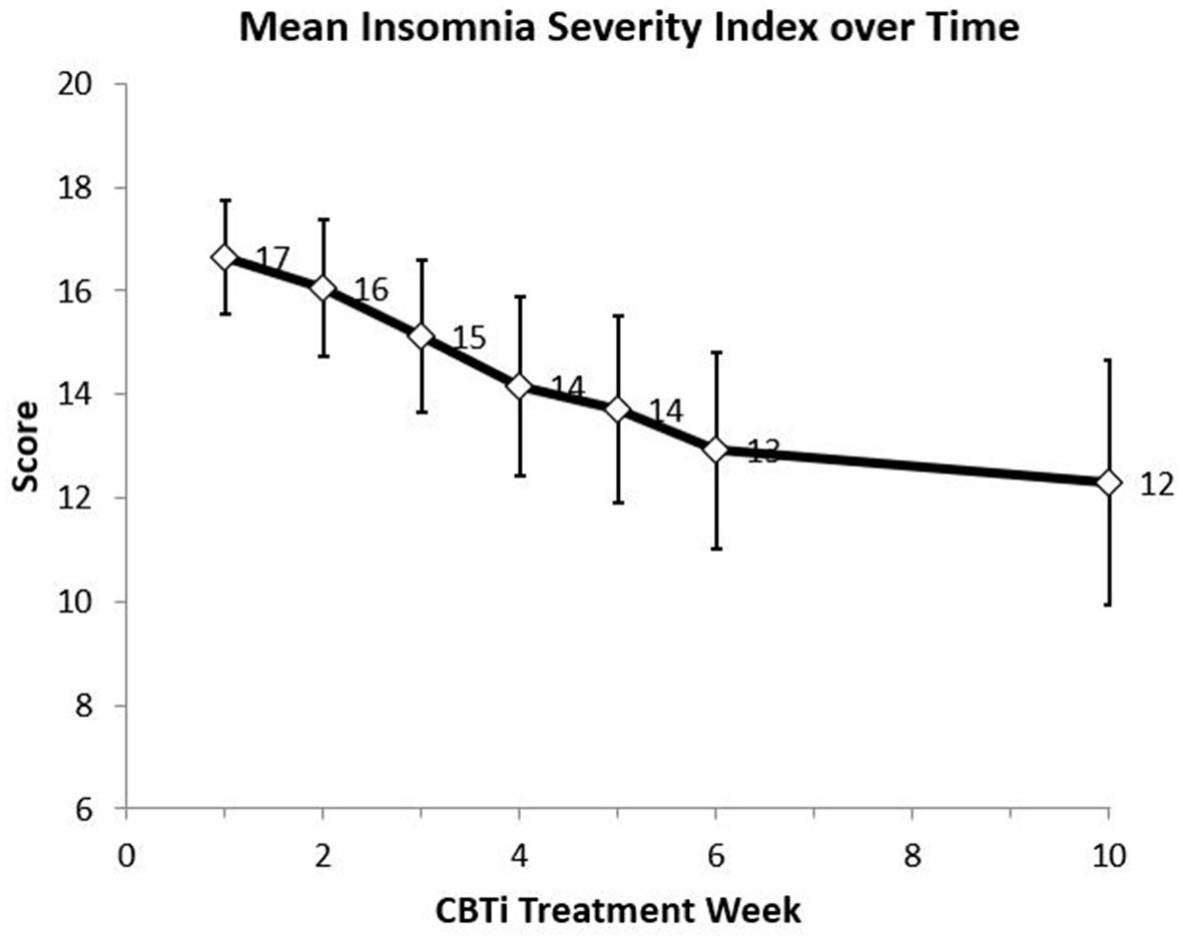
Mean Insomnia Severity Index (ISI) score over 10 weeks from initiation of CBTi intervention (Week 1 *N* = 69, Week 2 *N* = 60, Week 3 *N* = 56, Week 4 *N* = 54, Week 5 *N* = 51, Week 6 *N* = 45, Week 10 *N* = 36).

Mean levels for ISI also differed significantly over time (*p* < 0.001). The mean ISI dropped from a score of 17 at week 1 to 13 at week 6 and 12 at week 10 (*p* < 0.001 for pairwise comparisons vs. week 1) (Figure 1). No statistically significant changes on ESS scores were demonstrated among patients.

No statistically significant changes between week 6 and week 10 were found on any sleep diary or questionnaire outcome measure.

## 5 Discussion

Overall, results from this project found that patients who were being evaluated for TBIs at a military TBI clinic and completed CBTi improved during and after treatment on several sleep treatment outcomes. Improvements in treatment were found for SOL, WASO, and self-reported symptoms of insomnia.

To our knowledge, these findings are the first to demonstrate the utility of CBTi used as part of routine care to address insomnia among MHS beneficiaries being seen for evaluation and/or treatment of TBIs. Results from the current project are consistent with findings from earlier case studies and a clinical program evaluation, which found CBTi improved sleep for both individuals who have sustained TBIs as well as SMs in general (Ouellet and Morin, 2007; Lee et al., 2021). Further, our findings are consistent with studies that have found technology based CBTi skills programs are effective in addressing sleep problems among individuals who have sustained TBIs (Theadom et al., 2018).

Importantly, this project is one of the first to report on the utilization of a smartphone app to aid in the implementation of CBTi among patients being seen for TBIs. Specifically, these analyses found that many patients had chosen to use CBTi Coach to complete their sleep diaries as opposed to completing these using a pencil-and-paper format. Further, 67% of patients (24 of 36 patients) completed sleep diaries at their one-month follow-up session, after completing CBTi with their provider. Patients in the current project who stopped completing their sleep diaries by their post-treatment session cited low motivation and lack of accountability as the reason. The completion of sleep diaries in CBTi is critical to treatment effectiveness, as research has found that homework completion (e.g., sleep diaries) predicts insomnia remission following CBTi (Dong et al., 2018). Although smartphone apps used to improve sleep have been available for over a decade, there has been little research on whether such apps improve CBTi treatment adherence and treatment outcome (Koffel et al., 2018).

A 48% attrition rate (33 out of 69 patients) at a 1-month follow-up for the current evaluation is consistent with other studies examining CBTi (Vincent and Lewycky, 2009). Vincent and Lewycky (2009) examined the effectiveness of CBTi in a clinical setting among patients with insomnia and determined that 41.5% dropped out of treatment by their 1-month follow up session. Similarly, another study found that 42% of a clinical sample of SMs being seen for CBTi completed fewer than four sessions of the program (Lee et al., 2021).

This project has several limitations that warrant mention. Most importantly, these findings are limited as this was not a formal research study and was instead approved as a performance improvement project to determine whether an evidence-based treatment program for sleep was feasible in the context of a multidisciplinary care for TBIs. Therefore, the project is limited in terms of study design and data collection. For example, a control group was not used in this project. Therefore, conclusions on the effectiveness of CBTi for patients being seen in TBI clinics is limited. Further, sleep diary data was not verified by a second reviewer as data was collected entirely as part of each patient's clinical care. Lastly, this project evaluated MHS beneficiaries as an entire group and therefore did not examine treatment effectiveness among separate beneficiary subgroups such as SMs, family members, or retirees. Thus, conclusions on how military personnel responded to CBTi are limited. Despite that, anecdotal accounts from TBI clinic leadership indicate that SMs comprise of ~90% of all patients being seen in the clinic at any point in time.

Overall, findings demonstrate that CBTi, integrated with the CBTi Coach app, may be a promising treatment to address insomnia for those seeking care in TBI clinics and warrant further evaluation on the effectiveness of this treatment for individuals who have sustained TBIs, particularly among SMs. If effective, smartphone assisted CBTi may become a standard of care in military TBI programs.

## Data availability statement

The raw data supporting the conclusions of this article will be made available by the authors, without undue reservation.

## Ethics statement

The studies involving humans were approved by the Tripler Army Medical Center Human Protections Exempt Determinations Review Board. The studies were conducted in accordance with the local legislation and institutional requirements. Written informed consent for participation was not required from the participants or the participants' legal guardians/next of kin because Project was determined to be a quality improvement evaluation of a clinical program as part of routine clinical care and not a research study and only de-identified data was used.

## Author contributions

JM: Conceptualization, Data curation, Investigation, Methodology, Project administration, Supervision, Writing–original draft, Writing–review & editing. NK: Writing–original draft. ML: Formal analysis, Writing–original draft, Writing–review & editing. CC: Writing–review & editing. CG: Resources, Writing–review & editing.

## Footnotes

^1^Veterans Affairs Palo Alto Healthcare System (n.d.). *Cognitive Behavioral Therapy for Insomnia Group* [unpublished manuscript]. Psychology Service, Veterans Affairs Palo Alto Healthcare System.
